# Is Fear of COVID-19 Contagious? The Effects of Emotion Contagion and Social Media Use on Anxiety in Response to the Coronavirus Pandemic

**DOI:** 10.3389/fpsyg.2020.567379

**Published:** 2021-01-05

**Authors:** Michael G. Wheaton, Alena Prikhidko, Gabrielle R. Messner

**Affiliations:** ^1^Department of Psychology, Barnard College, Columbia University, New York, NY, United States; ^2^Department of Leadership and Professional Studies, School of Counseling, Recreation and School Psychology, Florida International University, Miami, FL, United States

**Keywords:** pandemic (COVID-19), media effect, anxiety, COVID-19, emotion contagion

## Abstract

The novel coronavirus disease (COVID-19) has become a global pandemic, causing substantial anxiety. One potential factor in the spread of anxiety in response to a pandemic threat is emotion contagion, the finding that emotional experiences can be socially spread through conscious and unconscious pathways. Some individuals are more susceptible to social contagion effects and may be more likely to experience anxiety and other mental health symptoms in response to a pandemic threat. Therefore, we studied the relationship between emotion contagion and mental health symptoms during the COVID-19 pandemic. We administered the Emotion Contagion Scale (ESC) along with a measure of anxiety in response to COVID-19 (modified from a previous scale designed to quantify fear of the Swine Flu outbreak) and secondary outcome measures of depression, anxiety, stress, and obsessive-compulsive disorder (OCD) symptoms. These measures were completed by a large (*n* = 603) student sample in the United States. Data were collected in the months of April and May of 2020 when the fear of COVID-19 was widespread. Results revealed that greater susceptibility to emotion contagion was associated with greater concern about the spread of COVID-19, more depression, anxiety, stress, and OCD symptoms. Consumption of media about COVID-19 also predicted anxiety about COVID-19, though results were not moderated by emotion contagion. However, emotion contagion did moderate the relationship between COVID-19-related media consumption and elevated OCD symptoms. Although limited by a cross-sectional design that precludes causal inferences, the present results highlight the need for study of how illness fears may be transmitted socially during a pandemic.

## Introduction

The novel coronavirus disease (COVID-19) is a viral respiratory infection that was identified in Wuhan, China in late 2019. COVID-19 was officially declared a pandemic on 11 March 2020 (World Health Organization, 2020) and has spread rapidly across the globe. The United States recently reached a grim milestone of 200,000 COVID-19-related deaths (CDC, 2020). COVID-19 has had a severe impact on healthcare systems, economic activity, and has caused widespread social disruption. The effects of COVID-19 on mental health and emotional well-being are likely to be enormous as well. A recent survey by the U.S. Census Bureau found that one-third of Americans are showing signs of clinical anxiety or depression related to COVID-19 (National Center for Health Statistics, 2020).

It is critical to study the factors that relate to impaired mental health and emotional symptoms in response to COVID-19 to understand how the public responds to pandemic illness threats. Existing research has investigated factors related to mental health symptoms in response to illness threats such as the H1N1 “Swine Flu” influenza (Tausczik et al., 2012; Wheaton et al., 2012), Zika virus (Blakey and Abramowitz, 2017), SARS (Xie et al., 2011), and Ebola (Blakey et al., 2015; Thompson et al., 2017). This work has largely suggested that pandemic illness threats are associated with widespread anxiety and worry among the public. Importantly, anxiety about one’s health can be helpful and adaptive in moderation, as it can focus attention and promote utilization of health-protective behaviors (e.g., handwashing and maintaining social distance). However, for some individuals, anxiety in response to a pandemic threat can become excessive and maladaptive (Asmundson and Taylor, 2020). Excessive anxiety can be debilitating and lead to severe impairment in functioning. In addition, some individuals may develop excessive behavioral responses to prevent infection. For example, past work (Brand et al., 2013) has linked anxiety about pandemic illnesses to extensive washing and cleaning compulsions, which are hallmark symptoms of obsessive-compulsive disorder (OCD), a psychiatric disorder that can be disabling when severe (American Psychiatric Association, 2013).

Research suggests that fear about an illness and associated behavioral changes can also spread virally (Asmundson and Taylor, 2020). For example, media reports about serious contagious illnesses can lead to threat-based reactions as has been shown historically, including in reports that link mass panic about the 1665 London plague to newspaper articles, prompting officials to shut down printing presses (Defoe, 2016). Similarly, consumption of media reports about more recent pandemic illness outbreaks has also been associated with anxiety symptoms (Xie et al., 2011; Tausczik et al., 2012). The mechanisms by which media reports lead to anxiety and other emotional reactions to a pandemic require further study. In particular, work is needed to determine which factors predispose certain individuals to experience these symptoms. One potential factor that has not received sufficient empirical attention is the construct of emotion contagion.

Emotion contagion, a construct from the field of social psychology, refers to the well-established finding that the emotion and affective behavior experienced by one individual may be influenced by that of others through conscious or unconscious pathways (Hatfield et al., 1993). For example, seeing other people cry at a funeral may provoke sadness and tearfulness in oneself. A large body of work in the field of social psychology supports the social transmission of emotion in laboratory experiments (Hatfield et al., 2014). Recent experimental work also suggests that emotional states can be transferred to others via social media (Kramer et al., 2014). Susceptibility to emotion contagion has been investigated as an individual difference variable, as some are more likely to take on social emotions than others. Evidence to support the notion that individuals differ in their tendency to be influenced by the emotions expressed by other people comes from data showing large individual differences in self-reported emotional contagion (Doherty, 1997; Sonnby–Borgström, 2002). In addition, self-reported susceptibility to emotion contagion has been linked to brain areas in the mirror neuron system for emotions (Lawrence et al., 2006; Pfeifer et al., 2007) as well as the ability to detect authentic facial emotions (Manera et al., 2013).

Several forms of emotions appear to be socially contagious, including happiness, anger, and anxiety (Behnke et al., 1994). However, data suggest that negative emotions are particularly transmissible among strangers (Paukert et al., 2008). Recent empirical work shows that individuals higher in emotion contagion have more stress reactivity in response to traumatic events (Trautmann et al., 2018). Thus, it is possible that people who are more in tune with the pervasive emotions of others may be particularly affected during the COVID-19 outbreak.

Whereas the majority of work on emotion contagion has focused on personal interactions, recent work has determined that emotions can be transmitted digitally (Coviello et al., 2014; Goldenberg and Gross, 2020). This is particularly important given that social media is ubiquitous in the present moment and many adults get their news through social media. As the virus has spread across the world, it has garnered significant media attention. Based on past research, this media exposure is likely to increase anxiety about COVID-19, but this effect might be especially strong for individuals higher in susceptibility to emotion contagion.

The present study sought to investigate the relationship between susceptibility to emotion contagion, media usage, and emotional responses to the COVID-19 outbreak in a large sample of adult residents of the United States during the early phase of the illness threat. We explored the novel hypothesis that individuals higher in emotion contagion would have heightened concerns about COVID-19 as well as more other mental health symptoms (anxiety, depression, stress, and OCD symptoms). Given that consumption of media about COVID-19 and utilization of social media may also heighten anxiety about the virus, we conducted a regression analysis controlling for these factors. Finally, we tested the possibility that emotion contagion might potentiate the relationship between media use and concern about COVID-19 via moderation analysis. As a secondary set of outcomes, we also evaluated other mental health variables including symptoms of depression, anxiety, stress, and OCD.

## Materials and Methods

### Participants

Participants (*n* = 603) were recruited from psychology classes at Florida International University (FIU) and were English-speaking residents of the United States. There were no other inclusion/exclusion criteria. The total sample consisted of 528 females (87.6%) and 71 males (11.8%). Three individuals (0.5%) selected “prefer not to say” when asked about gender identity and one respondent (0.2%) left the question blank. The sample had a mean age of 22.92 years (*SD* = 4.83, range 18−48). Race-ethnicity was assessed via two separate questions. On the first question, participants were asked to check a box to identify with a race: 63.0% selected “White/Caucasian,” 17.6% selected “Black/African American,” 4.1% selected “Asian/Pacific Islander,” 14.3% selected “other,” and 1.0% left the question blank. On a separate question, participants were asked “are you of Hispanic/Latino descent” and 74.3% selected yes.

### Procedure

Data were collected from 5 April to 13 May 2020, which comprised some of the early months of the COVID-19 outbreak in the United States. The FIU student sample was recruited from psychology classes and completed the survey in exchange for course credit. Data were collected via an online survey that was built using the online survey tool, Qualtrics. The first page of the survey comprised the study consent form. On the following pages, participants completed the set of self-report questionnaires listed below (in a fixed order), as well as a series of demographic questions. The study was reviewed and approved by the institutional review board (IRB).

### Measures

#### Emotion Contagion Scale (ECS; Doherty, 1997)

The ECS is a 15-item self-report scale that assesses the tendency to “catch” the emotions expressed by others. Items on the ECS assess susceptibility to the social transmission of five basic emotions: anger, fear, sadness, happiness, and love. Items are scored on five-point Likert scales from *not at all* (1) to *always* (5). The ECS has good psychometric properties and is commonly used in research studies. In the current study, the item stems for certain items on the scale were modified to include examples of how emotions could be transmitted online (e.g., “I cry at sad videos on social media”). In the present sample, the scale had good internal consistency (*α* = 0.88).

#### COVID Threat Scale (CTS)

The COVID-19-threat Scale is a nine-item, self-report inventory that was created for the present study by adapting a questionnaire developed to assess anxiety in response to the H1N1 “Swine Flu” influenza (Brand et al., 2013). Items utilize a five-point Likert Scale from *not at all* (1) to *very much* (5) to assess threat-perceptions of COVID-19. Items assess threat-related perceptions about the extent to which COVID-19 may spread across the United States, concerns about becoming ill or family members becoming ill, and changes in behavior (e.g., excessive handwashing). A tenth item assessing perception that COVID-19 would become a pandemic was not analyzed because COVID-19 was officially declared a pandemic by the WHO. The full scale appears in the appendix and analysis of the factor structure of the scale appears in the data supplement. Higher scores reflect greater anxiety and threat-related behaviors in response to COVID-19. The scale had acceptable internal consistency in this sample (*α* = 0.76).

#### Depression Anxiety Stress Scales 21 (DASS-21; Antony et al., 1998)

The DASS-21 is a short form of the original 42-item DASS (Lovibond and Lovibond, 1995). The scale is comprised of three separate subscales, measuring self-reported depression, anxiety, and stress on a 0−4 point scale. The DASS-21 subscales have strong evidence of reliability and construct validity (Henry and Crawford, 2005). The three subscales of the DASS demonstrated good internal consistency in the present study (range in *α*’s = 0.85−0.90).

#### Obsessive-Compulsive Inventory-Revised (OCI-R; Foa et al., 2002)

The OCI-R is an 18-item self-report questionnaire that assesses six dimensions of OCD symptoms: (a) washing, (b) checking/doubting, (c) obsessing, (d) neutralizing, (e) ordering, and (f) hoarding. Participants rate the degree to which they are bothered or distressed by OCD symptoms in the past month on a five-point scale from *not at all* (0) to *extremely* (4). OCI-R total scores have demonstrated excellent psychometric properties and validity (Foa et al., 2002). Reliability in the present sample was excellent (*α* = 0.92). Reliability for the OCI-R subscales was as follows: hoarding = 0.84, checking = 0.68, ordering = 0.87, ordering = 0.78, washing = 0.79, and obsessing = 0.84.

#### Social Media Utilization

For the purposes of the present study, participants were asked a simple question “How much time per day you spend on social media?” on a six-point ordinal response scale. Categories were scored as *not at all* (1), *a few minutes* (2), *about an hour* (3), *between 1 and 3 h* (4), *between 3 and 8 h* (5), *more than 8 h* (6). Similarly, participants were asked “How much time per day do you spend reading, watching or listening to news about the coronavirus?” which was answered on the same time per day response scale. The time frame for these questions was “recently” (i.e., “within the past week”).

### Statistical Analysis

We first computed correlation coefficients among the study measures to assess the relationship between emotion contagion, media use, and the primary (concerns about COVID-19) and secondary outcomes (anxiety, depression, stress, and OCD symptoms). Next, we tested the hypothesis that emotion contagion would predict the degree of fear of COVID-19 controlling for exposure to COVID-19 media and social media utilization. To do this, we entered the ECS scores, social media consumption (per day), and COVID-19-media consumption as predictor variables in a simultaneous regression predicting concern about COVID-19, as indexed by CTS scores. Finally, we tested the possibility that emotion contagion might potentiate the relationship between media use and concern about COVID-19 via moderation analysis conducted through the PROCESS SPSS macro (Preacher and Hayes, 2008). Subsequent supplementary analyses similarly tested for moderation effects among the secondary outcomes (anxiety, depression, stress, and OCD symptoms). Finally, as a sensitivity analysis, we tested both whether scores on study measures differed by study enrollment time period via independent samples *t*-tests and whether enrollment period affected primary regression results. Statistical analyses were run using the IBM SPSS (Version 23, Armonk, NY, United States).

## Results

### Correlations

Table 1 presents the correlations among study measures. As shown in the table, greater susceptibility to emotion contagion was significant and positively correlated with concern about COVID-19 (*r* = 0.32, *p* < 0.001), depression (*r* = 0.12, *p* < 0.01), anxiety (*r* = 0.27, *p* < 0.001), stress (*r* = 0.29, *p* < 0.001), and OCD symptoms (*r* = 0.29, *p* < 0.001). Emotion contagion susceptibility was weakly yet significantly correlated with daily social media consumption (*r* = 0.13, *p* < 0.01) and consumption of media related to COVID-19 (*r* = 0.12, *p* < 0.01). Similarly, concern about COVID-19 was also weakly yet significantly correlated with hours per day of self-reported daily social media consumption (*r* = 0.11, *p* < 0.01) and consumption of media related to COVID-19 (*r* = 0.21, *p* < 0.001).

**TABLE 1:** Correlations among study measures.

	ECS	CTS	DASS-D	DASS-A	DASS-S	OCI-R	Time/day COVID-19	Time/day social media
ECS	–							
CTS	0.32**	–						
DASS-D	0.12*	0.17**	–					
DASS-A	0.27**	0.23**	0.71**	–				
DASS-S	0.29**	0.28**	0.78**	0.79**	–			
OCI-R	0.29**	0.24**	0.53**	0.59**	0.58**	–		
Time/day COVID-19	0.12*	0.21**	0.14*	0.21**	0.17**	0.17**	–	
Time/day social media	0.13*	0.11*	0.14*	0.14*	0.15**	0.12*	0.18**	–

* P < 0.01.** P < 0.001.Note. ECS, Emotion Contagion Scale; CTS, COVID-19 Threat Scale; DASS, Depression Anxiety Stress Scale; OCI-R, Obsessive-Compulsive Inventory-Revised.

### Regression Results

COVID Threat Scale scores were slightly negatively skewed (−0.75) and leptokurtic (0.87) but did not violate normality assumptions for regression analysis (skew and kurtosis < 1). Regression diagnostics indicated no problems with multicollinearity; the data met the assumption of independent errors (Durbin–Watson value = 2.05) and the histogram of standardized residuals indicated that the data contained approximately normally distributed errors, as did the normal P-P plot of standardized residuals. The data also met the assumptions of homogeneity of variance and linearity.

Results showed that the overall model (which included gender, time per day using social media, consumption of COVID-19 articles, and ECS scores) accounted for 14% of the variance in concerns about COVID-19, which was significant (*R*^2^ = 0.14, *p* < 0.001). Inspection of the individual regression coefficients revealed that the ECS was a significant individual predictor of concerns about COVID-19 [*b* = *0*.14 (*SE* = 0.02), *p* < 0.001], as was time per day consuming articles about COVID-19 [*b* = *0*.83 (*SE* = 0.20), *p* < 0.001]. Time per day using social media was not a significant predictor in the model [*b* = *0*.17 (*SE* = 0.19), *p* = 0.37], nor was participant gender [*b* = *0*.50 (*SE* = 0.63), *p* = 0.43].

### Moderation Effects

We explored the possibility that emotion contagion might moderate the relationship between excessive concern about COVID-19 and both general social media utilization and COVID-19-related media consumption. Separate analyses were run considering the two media utilization questions as independent variables.

In the first regression model, concern about COVID-19 (CTS scores) was set as the dependent variable, daily consumption of media pertaining to COVID-19 was set as the independent variable, and emotion contagion was set as the moderator. Results found significant main effects for emotion contagion [*b* = *0*.86 (*SE* = 0.19), *p* < 0.001] and COVID-19-related media consumption [*b* = *0*.14 (*SE* = 0.02), *p* < 0.001] but the interaction term was not significant [*b* = −0.01 (*SE* = 0.02), *p* = 0.51].

In the second regression model, concern about COVID-19 (CTS scores) was set as the dependent variable, daily utilization of social media was set as the independent variable, and emotion contagion was set as the moderator. Results found significant main effects for emotion contagion [*b* = *0*.15 (*SE* = 0.02), *p* < 0.001]. The main effect for daily use of social media was not significant [*b* = *0*.30 (*SE* = 0.19), *p* = 0.13] and the interaction term was not significant [*b* = −0.002 (*SE* = 0.02), *p* = 0.88].

### Supplementary Analyses

**FIGURE 1:**
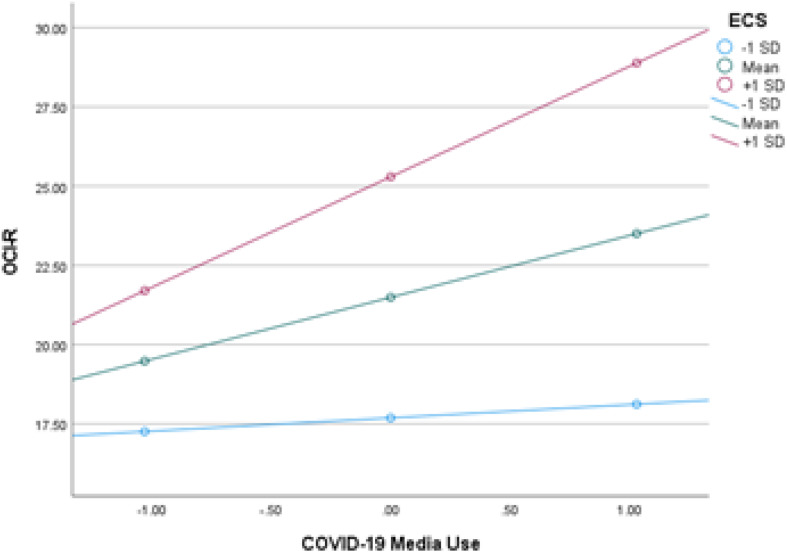
Emotion contagion as a moderator of the association between COVID-19 media and OCD symptoms. *Note*. ECS, Emotion Contagion Scale; OCI-R, Obsessive-Compulsive Inventory-Revised.

As a secondary analysis, we explored predictors of the other mental health outcome variables (DASS-21 subscales and OCI-R scores). These analyses are presented in full in the Supplementary Material. Overall results showed that emotion contagion was an independent predictor of each of the DASS-21 subscales and of OCI-R scores. Daily consumption of information related to COVID-19 also predicted all secondary outcomes, while daily time on social media predicted DASS-21 Depression and DASS-21 Stress but not OCI-R or DASS-21 Anxiety. There was only one significant moderator: ECS scores moderated the relationship between consumption of COVID-19 information and OCI-R scores such that the strength of this association increased along with increasing ECS scores (see Figure 1). Analysis of OCI-R subscale scores revealed that emotion contagion predicted each of the OCI-R subscales. Moreover, ECS scores moderated the connection between consumption of COVID-19 media and four OCI-R subscales (washing, checking, neutralizing, and hoarding), as well as the connection between daily social media use and two OCI-R subscales (obsessing and hoarding) suggesting the emotion contagion may be more relevant for the connection between specific OCD symptoms and media use domains (see Supplementary Material for details).

### Analysis by Study Enrollment Period

Given that data collection spanned from 5 April to 13 May 2020, we investigated whether responses varied by date of enrollment. The median response date was 13 April and we divided the participants based on whether they had completed the survey in the first or second half of responses. The two groups of participants (early vs. late responders) did not differ in terms of time per day on social media (*t* = −0.81, *p* = 0.42), consumption of media about COVID-19 (*t* = −1.04, *p* = 0.30), or CTS scores (*t* = −0.72, *p* = 0.47). Regression results predicting CTS scores were similar in both enrollment periods (see Supplementary Material). These results suggest that concerns about COVID-19 were stable during the study period, and may have become widespread even before data collection began. Similarly, scores on the DASS-21 subscales and OCI-R did not differ by enrollment period (all *p*’s > 0.26).

## Discussion

We investigated emotional responses during the COVID-19 pandemic outbreak in the United States in a large student sample and tested the possibility that individuals with greater susceptibility to emotion contagion would experience more distress and symptomatic behavior. Results were in line with our hypothesis: individuals with greater levels of susceptibility to emotion contagion had greater levels of anxiety about COVID-19, more depression, anxiety, and stress, and greater levels of OCD symptoms. These findings are discussed in detail below.

Although the magnitude of the associations was modest, our overall findings suggested that individual differences in susceptibility to emotion contagion tracked with emotional symptoms, such as anxiety about the virus and maladaptive behavioral responses (i.e., OCD symptoms). This finding is in line with past work showing that individuals higher in emotion contagion experience more stress responses to traumatic events (Trautmann et al., 2018). In this case, the COVID-19 outbreak itself represents a form of community stressor, causing widespread anxiety and worry in the public. Those high in emotion contagion are likely more attuned to the emotional experiences of others and thereby experience heightened anxiety.

We also considered the possibility that general social media use and consumption of media about COVID-19 in particular would also be relevant to predicting degree of anxiety around COVID-19. Furthermore, we considered that emotion contagion might act as a moderator for these relationships. Results showed that consumption of media about COVID-19 significantly predicted degree of COVID-19-related anxiety but this relationship was not moderated by emotion contagion. One prior report found that degree of emotion contagion moderated stress response following exposure to a traumatic film but this took place under laboratory conditions and with a highly controlled stressor (Trautmann et al., 2018), whereas our measure of exposure to media on COVID-19 was retrospective and uncontrolled. Therefore, it is possible that under more controlled conditions, a significant moderation effect would have emerged.

Alternatively, it is possible that the link between emotion contagion and fear of a pandemic is independent of exposure to media articles, as other pathways may connect these variables (e.g., information provided via family and friends). Nevertheless, our results linked both media consumption and emotion contagion to concern about COVID-19. This result is in line with research on past pandemics, which found positive associations between consuming media reports about the outbreak and anxiety (Xie et al., 2011; Tausczik et al., 2012). We also found that susceptibility to emotion contagion was related to concern about the spread of COVID-19, as well as other mental health outcomes (depression, anxiety, stress, and OCD symptoms). Emotion contagion was somewhat more strongly associated with concern about COVID-19, anxiety, and stress, and less strongly linked with depression, which may relate to anxiety being the predominant emotion experienced by the public during this time (National Center for Health Statistics, 2020).

In our supplemental analysis, we did find that the link between consumption of media about COVID-19 and OCD symptoms was significantly moderated by emotion contagion such that this association was stronger for individuals higher in emotion contagion. To the extent that OCD symptoms (e.g., compulsive washing and cleaning) might relate to the virus that causes COVD-19, it is possible that proneness to socially transmitted emotions enhanced the link between consumption of alarming articles about COVID-19 and increased OCD symptoms. However, caution is warranted in interpreting this finding because it was conducted as a secondary analysis and the cross-sectional nature of the data preclude drawing causal inferences. In addition, our sample was non-clinical, and therefore, future research is needed in clinical populations.

Hours per day of social media use weakly yet significantly related to concern about COVID-19, but this relationship did not reach significance in our regression model controlling for gender and consumption of COVID-19 related media. Among the secondary outcomes, time interacting with social media did predict symptoms of depression and stress, but not anxiety or OCD symptoms. Emotion contagion did not moderate the link between social media use and the mental health outcome variables. This result suggests that simply using social media may not robustly be linked with all mental health outcomes during a pandemic threat. Rather it may be important to consider the content of social media with which one interacts. Emerging work has suggested that the mental health effects of social media use may be highly variable, with some forms of social media use linked to deleterious mental health outcomes, while other social media use (e.g., that which encourages belongingness and social connection) may improve mental health outcomes (Clark et al., 2018). Thus, future research with more fine-grained analysis of social media utilization is needed.

## Limitations

Present results should be interpreted in light of several important study limitations. First, all data were collected online utilizing a cross-sectional design in which participants completed measures at a single time point. Thus, our results are not able to firmly establish cause and effect relationships. For example, we cannot determine whether media exposure increased anxiety or whether individuals with heightened anxiety were more likely to seek information and therefore spent more time engaging with media. A more powerful design would be a longitudinal study to follow individuals low and high in emotion contagion to compare their utilization of social media and trajectories for anxiety and stress in response to a pandemic threat. Similarly, other variables (such as neuroticism) should be considered as potential third variables explaining the associations between observed relationships. Unfortunately, neuroticism was not assessed in our survey, representing an important future direction for research. In addition, all data were collected via self-report surveys, and reliance on self-report questionnaires may have inflated the relationship between variables due to shared methods variance. Future study using mixed methods approaches including interviews would add methodological diversity to measurement. Finally, the data were limited to a student sample (that was mostly female). Therefore, future research is needed to replicate these findings in other samples, including among clinical samples of individuals experiencing anxiety disorders who may be particularly affected by fears of pandemic illness threats (Dennis et al., 2020), as well as samples with greater numbers of male participants. Given that the results of some of the present analyses were statistically significant but of small magnitude, their clinical significance requires further study.

## Conclusion

Notwithstanding these limitations, the present report highlights the possibility that emotion contagion effects may contribute to emotional reactions during a pandemic illness outbreak, such as COVID-19. We found that those who were higher in susceptibility to emotion contagion experienced more concern about the spread of COVID-19, more anxiety, stress, and depression and greater OCD symptoms. Together, these data suggest that maladaptive emotional experiences may be socially contagious during a pandemic threat.

## Data Availability Statement

The raw data supporting the conclusions of this article will be made available by the authors, without undue reservation.

## Ethics Statement

The studies involving human participants were reviewed and approved by the Institutional Review Board at Florida International University. The participants provided their written informed consent to participate in this study.

## Author Contributions

MW and AP conceived of the idea for this study. AP sought and received approval from the local IRB and coordinated data collection. MW and GM conducted the statistical analysis. All authors contributed to the final write up and all reviewed and approved the submission.

## Conflict of Interest

The authors declare that the research was conducted in the absence of any commercial or financial relationships that could be construed as a potential conflict of interest.

## Appendix

**TABLE TA1:** Covid-19 Threat Scale (CTS).

	NOT AT ALL	A LITTLE	SOME	MUCH	VERY MUCH
1. To what extent are you concerned about Coronavirus?	1	2	3	4	5
2. How likely is it that you could become infected with Coronavirus?	1	2	3	4	5
3. How likely is it that someone you know could become infected with Coronavirus?	1	2	3	4	5
4. How quickly do you believe contamination from Coronavirus is spreading in the U.S.?	1	2	3	4	5
5. How much exposure have you had to information about Coronavirus?	1	2	3	4	5
6. If you did become infected with Coronavirus, to what extent are you concerned that you will be severely ill?	1	2	3	4	5
7. To what extent has the threat of Coronavirus influenced your decisions to be around people?	1	2	3	4	5
8. To what extent has the threat of Coronavirus influenced your travel plans?	1	2	3	4	5
9. To what extent has the threat of Coronavirus influenced your use of safety behaviors (e.g., wearing a mask in public or using hand sanitizer)?	1	2	3	4	5
