# Supplementary material for: Is Fear of COVID-19 Contagious? The Effects of Emotion Contagion and Social Media Use on Anxiety in Response to the Coronavirus Pandemic
Source: Front Psychol. 2021 Jan 5;11:567379. doi: 10.3389/fpsyg.2020.567379 (PMC7813994; doi:10.3389/fpsyg.2020.567379)
Supplement: Supplementary file 1 [file Data_Sheet_1.docx]

Supplementary Material

# Factor Analysis of the COVID Threat Scale (CTS)

As recommended by a Reviewer, we examined the factor structure of the COVID-19 Threat Scale (CTS). We submitted the 9 items to an exploratory factor analysis (EFA) with oblique (promax) rotation to allow the factors to be correlated. The decision of how many factors to retain was based on examination of the eigenvalues and scree plot. The following results were obtained:

| Component | Initial Eigenvalues | | |
| --- | --- | --- | --- |
|  | Total | % of Variance | Cumulative % |
| 1 | 3.230 | 35.885 | 35.885 |
| 2 | 1.352 | 15.024 | 50.909 |
| 3 | .922 | 10.244 | 61.153 |
| 4 | .790 | 8.781 | 69.933 |
| 5 | .692 | 7.685 | 77.618 |
| 6 | .620 | 6.893 | 84.511 |
| 7 | .538 | 5.981 | 90.492 |
| 8 | .490 | 5.449 | 95.942 |
| 9 | .365 | 4.058 | 100.000 |


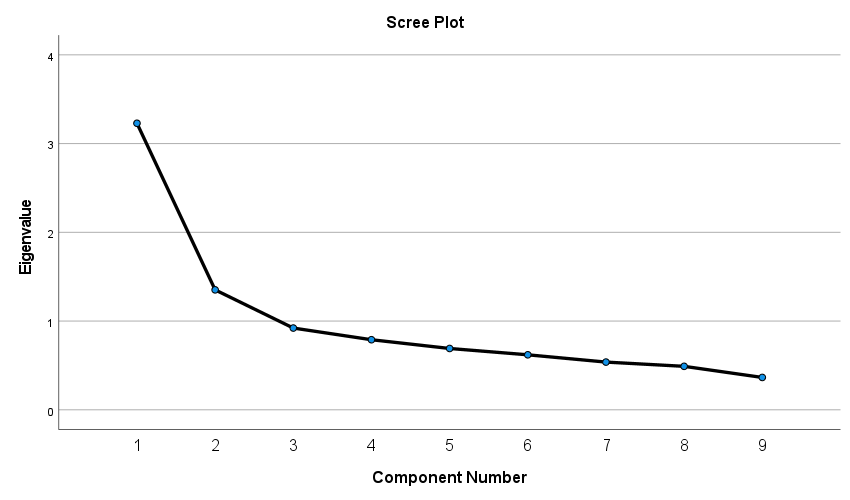


Based on the results we examined a 2-factor solution, in which the items had the following loadings:

| **Pattern Matrix^a^** | | |
| --- | --- | --- |
|  | Component | |
|  | 1 | 2 |
| Please, select the answer that best describes how you have felt in the past week. - To what extent are you concerned about the Corona virus? | .654 | .099 |
| Please, select the answer that best describes how you have felt in the past week. - How likely it is that you could become infected with the Corona virus? | -.100 | .914 |
| Please, select the answer that best describes how you have felt in the past week. - How likely it is that anyone you know could become infected with the Corona virus? | .028 | .859 |
| Please, select the answer that best describes how you have felt in the past week. - How likely do you believe contamination from the Corona virus could spread in the U.S.? | .527 | .270 |
| Please, select the answer that best describes how you have felt in the past week. - How much exposure have you had to information about the Corona virus? | .371 | .235 |
| Please, select the answer that best describes how you have felt in the past week. - If you did become infected with the Corona virus, to what extent are you concerned that you will be severely ill? | .418 | .250 |
| Please, select the answer that best describes how you have felt in the past week. - To what extent has the threat of the Corona virus influenced your decisions to be around people? | .828 | -.144 |
| Please, select the answer that best describes how you have felt in the past week. - To what extent has the threat of the Corona virus influenced your travel plans? | .586 | -.037 |
| Please, select the answer that best describes how you have felt in the past week. - To what extent did the threat of the Corona virus influence behaviors, including wearing a mask or using hand sanitizer? | .810 | -.134 |
| Extraction Method: Principal Component Analysis.  Rotation Method: Promax with Kaiser Normalization. | | |
| a. Rotation converged in 3 iterations. | | |

Examination of the factor loadings revealed that items #2 and #3 loaded on the second factor whereas the other 7 items loaded on the first factor. The emergence of the second factor is likely due to the highly related content of item 2 (perceived likelihood of personally catching COVID-19) and item 3 (perceived likelihood of others catching COVID-19), which could represent a point of local dependence in the factor solution. Recommendations for subscale retention and scale development (Thurstone, 1947; Guadagnoli & Velicer, 1988) suggest that subscales with fewer than three items are less reliable and should not be retained. Therefore, we elected to retain analysis of the total score of the CTS. Further psychometric analysis of the scale revealed that each item had a corrected item-total correlation >.35 and internal consistency of the total score was acceptable (Chronbach’s alpha=.76). Analysis of the total score is consistent with how prior versions of these questions had been scored when applied to past pandemics (e.g., total scores on the Swine Flu Inventory in Wheaton et al., 2012 and Brand et al., 2013 which used the same items about Swine Flu).

**2. Analysis of secondary outcomes.**

Our primary aim was to explore emotion contagion as it relates to fears of COVID-19 (indexed by the CTS). However, as recommended by a Reviewer we conducted supplementary analyses to explore the relationship between emotion contagion and other mental health outcome variables (DASS-21 subscales and OCI-R scores). These analyses were conducted similarly to the primary outcome (CTS scores): First we predicted each outcome variable using the ECS, social media use questions, and participant gender. Next, moderation analysis was conducted using the PROCESS SPSS Macro (Preacher & Hayes, 2008) considering the two media utilization questions as independent variables and the ECS as a potential moderator. As these analyses were not part of our pre-planned hypothesis concerning fear of COVID-19, we considered these analyses to be an exploratory supplement to our original analysis. Results are organized by outcome variable below:

*DASS-21 Depression Subscale*

The regression model predicting DASS-21 Depression was significant (*R^2^*=.04, *p*<.001). Inspection of the individual regression coefficients revealed that the ECS was a significant individual predictor (*b=*.10 [*SE*=.04]*, p*=.022), as were both time per day consuming articles about COVID-19 (*b=*1.20 [*SE*=.44]*, p*=.007) and time per day using social media (*b=*1.15 [*SE*=.11]*, p*=.008). Participant gender was not a significant predictor (*b=-*.55 [*SE*=1.41]*, p*=.70).

Moderation analysis found that the interaction term for ECS X daily consumption of media pertaining to COVID-19 was not significant (*b=*.06[*SE*=.03]*, p*=.08) and nor was the interaction term for ECS X daily utilization of social media (*b=*.05[*SE*=.03]*, p*=.16).

*DASS-21 Anxiety Subscale*

The regression model predicting DASS-21 Anxiety scores was significant (*R^2^*=.11, *p*<.001). Inspection of the individual regression coefficients revealed that the ECS was a significant individual predictor (*b=*.22 [*SE*=.04]*, p*<.001), as was time per day consuming articles about COVID-19 (*b=*1.60 [*SE*=.39]*, p*<.001). Time per day using social media (*b=*.64 [*SE*=.38]*, p*=.09) and participant gender were not significant predictors (*b=*1.32 [*SE*=1.23]*, p*=.28).

Moderation analysis found that the interaction term for ECS X daily consumption of media pertaining to COVID-19 was not significant (*b=*.05[*SE*=.03]*, p*=.11) and nor was the interaction term for ECS X daily utilization of social media (*b=*.05[*SE*=.03]*, p*=.12).

*DASS-21 Stress Subscale*

The regression model predicting DASS-21 Stress was significant (*R^2^*=.11, *p*<.001). Inspection of the individual regression coefficients revealed that the ECS was a significant individual predictor (*b=*.24 [*SE*=.04]*, p*<.001), as were both time per day consuming articles about COVID-19 (*b=*1.18 [*SE*=.41]*, p*=.004) and time per day using social media (*b=*.83 [*SE*=.40]*, p*=.038). Participant gender was not a significant predictor (*b=*2.27 [*SE*=1.30]*, p*=.08).

Moderation analysis found that the interaction term for ECS X daily consumption of media pertaining to COVID-19 was not significant (*b=*.02[*SE*=.03]*, p*=.47) and nor was the interaction term for ECS X daily utilization of social media (*b=*.04[*SE*=.03]*, p*=.16).

*OCI-R*

The regression model predicting OCI-R scores was significant (*R^2^*=.11, *p*<.001). Inspection of the individual regression coefficients revealed that the ECS was a significant individual predictor (*b=*.38 [*SE*=.06]*, p<*.001), as was time per day consuming articles about COVID-19 (*b=*1.91 [*SE*=.58]*, p*=.001). Time per day using social media (*b=*.84 [*SE*=.56]*, p*=.14) and participant gender were not significant predictors (*b=-*2.70 [*SE*=1.84]*, p*=.88).

Moderation analysis found that the interaction term for ECS X daily hours of social media use was not significant (*b=*.06[*SE*=.04]*, p*=.19). However, the interaction term for ECS X daily consumption of media pertaining to COVID-19 was significant (*b=*.14[*SE*=.05]*, p*=.002). This significant interaction was probed by calculating the predictive ability of consumption of media articles for individuals at mean, high and low levels of emotion contagion (mean, +/- 1 SD on the ECS). Results showed that for individuals low in emotion contagion (-1 SD on the ECS), there was not a significant effect between COVID-19 related media consumption and OCI-R scores (*b=*.42[*SE*=.77]*, p*=.59). At the mean level of the ECS the effect was significant (*b=*1.95 [*SE*=.57]*, p*=.001), while the strength of this relationship was strongest for individuals at high in emotion contagion (+ 1 SD), (*b=*3.48[*SE*=.74]*, p*<.001). As shown in the Figure below, the positive relationship between consumption of media related to COVID-19 and OCI-R scores increased for individuals higher in ECS.


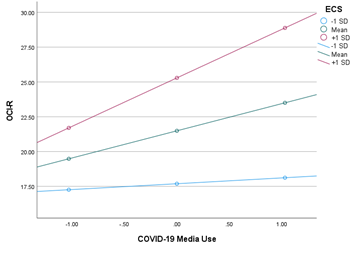


**3. Gender Effects**

As recommended by a Reviewer, we examined gender differences in emotion contagion susceptibility and responses to COVID-19. On the ECS, an independent samples *t*-test revealed that women (M=49.70, SD=10.17) had significantly higher scores as compared to men (M=40.21, SD=9.55), *t*=7.43, *p*<.001 indicating greater proneness to emotion contagion. In addition, similar to responses to past pandemics (Taylor, 2019) we found that women (M=35.54, SD=5.08) had higher scores than men (M=33.49, SD=5.70) on the CTS, *t*=3.14, *p*=.002, indicating greater threat responses to COVID-19. Importantly, the relationship between ECS and CTS was significant and of similar magnitude among both women (r=.29, *p*<.001) and men (r=.33, *p*=.005). Gender was added as a covariate to our regression analyses.

Regarding our secondary outcome measures (DASS-21 subscales and OCI-R scores), female participants had higher scores on DASS-21 Anxiety and DASS-21 Stress, but there were no significant gender differences on DASS-21 Depression or OCI-R scores as shown in the Table below:

| Measure | Female Mean (SD) | Male Mean (SD) | Test of the difference |
| --- | --- | --- | --- |
| DASS-21 Depression | 12.00 (11.02) | 11.75 (10.33) | *t*=0.18, *p*=.86 |
| DASS-21 Anxiety | 10.47 (10.14) | 6.99 (7.18) | *t*=3.63, *p*<.001 |
| DASS-21 Stress | 15.13 (10.67) | 10.20 (8.44) | *t*=3.37, *p*<.001 |
| OCI-R | 21.99 (15.07) | 18.48 (12.27) | *t*=1.88, *p*=.06 |

Analysis of the relationship between these secondary outcomes and the ECS is shown in the Table below. As shown, these correlations were significant only in female participants, suggesting that women may have driven the association between ECS and secondary outcomes in our sample. Therefore, future study of gender effects related to emotion contagion is needed, particularly in samples with more male participants.

| Measure | Correlation with ECS among Female participants | Correlation with ECS among Male participants |
| --- | --- | --- |
| DASS-21 Depression | *r*=.13, *p=*.002 | *r*=-.08, *p*=.52 |
| DASS-21 Anxiety | *r*=.25, *p*<.001 | *r*=.12, *p*=.32 |
| DASS-21 Stress | *r*=.27, *p*<.001 | *r*=.03, *p*=.78 |
| OCI-R | *r*=.30, *p*<.001 | *r*=-.02, *p*=.89 |

**4. Enrollment period effects**

As recommended during the review process, we sought to explore whether primary regression results differed by enrollment period. The median response date was April 13 and we divided participants based on whether they had completed the survey in the first or second half of responses. We then re-ran our primary regression analyses in the early and late responders separately. As shown below, regression results were the same for both enrollment periods.

Among the early enrollment period the regression model predicting CTS scores was significant (*R^2^*=.14, *p*<.001). Inspection of the individual regression coefficients revealed that the ECS was a significant individual predictor (*b=*.13 [*SE*=.03]*, p*<.001), as was time per day consuming articles about COVID-19 (*b=*1.03 [*SE*=.28]*, p*<.001). Time per day using social media was not a significant predictor (*b=.*14 [*SE*=.29]*, p*=.64). Results were nearly identical in the later enrollment period: the regression model predicting CTS scores was significant (*R^2^*=.14, *p*<.001) and the ECS (*b=*.16 [*SE*=.03]*, p*<.001) and time per day consuming articles about COVID-19 (*b=*.69 [*SE*=.28]*, p*=.01) were significant individual predictors whereas time per day using social media was not a significant predictor (*b=.*28 [*SE*=.25]*, p*=.28). To compare the predictive strength of the ECS by enrollment wave we computed squared semi-partial correlations in these regression models, which indicate unique variance in CTS accounted for by ECS scores. In the early enrollment period squared semi-partial correlations indicated that the ECS accounted for 7.29% of the variance in CTS scores, whereas in the late enrollment period the ECS accounted for 10.2% of variance in CTS scores. Thus, the pattern of results was similar in both enrollment periods (with the ECS emerging as a significant predictor in both phases), although emotion contagion may have been slightly more predictive of concern about COVID-19 in the second half of the enrollment period.

We also explored whether results of the moderation analyses would differ by study enrollment period. First, we tested whether ECS moderated the relationship between overall social media use and CTS score. Results showed that the interaction term was not significant in either the early (*b=*-.02 [*SE*=.02]*, p*=.26) or late study enrollment periods (*b=*.01 [*SE*=.02]*, p*=.49). We also tested whether the ECS moderated the link between CTS scores and consumption of media pertaining to COVID-19 specifically. Results showed that the interaction term was not significant in either the early (*b=*-.02 [*SE*=.02]*, p*=.28) or late (*b=*.01 [*SE*=.02]*, p*=.97) enrollment periods. These results suggest that concerns about COVID-19 were stable during the study period and that the relationship between these concerns and emotion contagion did not vary for by enrollment period.

**5. OCI-R subscale analyses**

As recommended during the review process, we explored results related to the OCI-R subscales in addition to the total score. The overall pattern of correlations was that they were strongest for the OCI-R washing subscale.

|  | OCI-R Total | OCI-R Hoarding | OCIR-R Checking | OCI-R Ordering | OCI-R Neut | OCI-R Washing | OCI-R Obsessing |
| --- | --- | --- | --- | --- | --- | --- | --- |
| CTS | .24** | .12** | .19** | .19** | .06 | .28** | .20** |
| DASS-D | .53** | .45** | .38** | .34** | .34** | .39** | .64** |
| DASS-A | .59** | .46** | .47** | .38** | .41** | .45** | .64** |
| DASS-S | .58** | .49** | .43** | .41** | .34** | .46** | .64** |
| ECS | .29** | .22** | .24** | .20** | .18** | .26** | .23** |
| Time/day COVID-19 | .17^**^ | .12** | .20** | .09* | .14** | .14** | .14** |
| Time/day social media | .12** | .15* | .07 | .06 | .04 | .10* | .13** |

**P*<.01

***P*<.001

Note. ECS=Emotion Contagion Scale; CTS=COVID-19 Threat Scale; DASS=Depression Anxiety Stress Scale; OCI-R=Obsessive-Compulsive Inventory-Revised.

Regression analyses

Regression analyses of the OCI-R subscales were conducted similarly to the other outcome variables. First, we predicted each OCI-R Subscale using the ECS, media use questions, and participant gender. Next, moderation analysis was conducted using the PROCESS SPSS Macro (Preacher & Hayes, 2008) considering the two media utilization questions as independent variables and the ECS as a potential moderator. Results are organized by subscale below:

*OCI-R Hoarding*

The regression model predicting OCI-R Hoarding scores was significant (*R^2^*=.07, *p*<.001). Inspection of the individual regression coefficients revealed that the ECS was a significant individual predictor (*b=*.06 [*SE*=.01]*, p<*.001), as was time per day using social media (*b=*.33 [*SE*=.12]*, p*<.01). Time per day consuming articles about COVID-19 (*b=*.22 [*SE*=.12]*, p*=.08) and participant gender (*b=-*.16 [*SE*=0.39]*, p*=.67) were not significant predictors.

Moderation analysis found that the interaction term for ECS X daily hours of social media use was significant (*b=*.02[*SE*=.01]*, p*=.034). Specifically, for individuals low in emotion contagion (-1 SD on the ECS), there was not a significant effect between daily social media use and OCI-R hoarding scores (*b=*.17[*SE*=.15]*, p*=.24). At the mean level of the ECS the effect was significant (*b=*.36 [*SE*=.12]*, p*=.002), while the strength of this relationship was strongest for individuals at high in emotion contagion (+ 1 SD), (*b=*.58[*SE*=.15]*, p*<.001). Similarly, the interaction term for ECS X daily consumption of media pertaining to COVID-19 was also significant (*b=*.03[*SE*=.01]*, p*=.002). For individuals low in emotion contagion (-1 SD on the ECS), there was not a significant effect between COVID-19 media consumption and OCI-R hoarding scores (*b=*-.06 [*SE*=.16]*, p*=.69). At the mean level of the ECS the effect was significant (*b=*.24 [*SE*=.12]*, p*=.044), while the strength of this relationship was strongest for individuals at high in emotion contagion (+ 1 SD), (*b=*.57 [*SE*=.15]*, p*<.001).

*OCI-R Checking*

The regression model predicting OCI-R Checking scores was significant (*R^2^*=.09, *p*<.001). Inspection of the individual regression coefficients revealed that the ECS was a significant individual predictor (*b=*.06 [*SE*=.01]*, p<*.001), as was time per day consuming articles about COVID-19 (*b=*.46[*SE*=.11]*, p*=.001). Time per day using social media (*b=*.02 [*SE*=.11]*, p*=.83) and participant gender (*b=*.24[*SE*=.35]*, p*=.49) were not significant predictors.

Moderation analysis found that the interaction term for ECS X daily hours of social media use was not significant (*b=*.01[*SE*=.01]*, p*=.74). However, the interaction term for ECS X daily consumption of media pertaining to COVID-19 was significant (*b=*.03[*SE*=.01]*, p*<.01). For individuals low in emotion contagion (-1 SD on the ECS), there was not a significant effect between COVID-19 related media consumption and OCI-R Checking scores (*b=*.19[*SE*=.14]*, p*=.19). At the mean level of the ECS the effect was significant (*b=*.44 [*SE*=.11]*, p*<.001), while the strength of this relationship was strongest for individuals at high in emotion contagion (+ 1 SD), (*b=*.72[*SE*=.14]*, p*<.001).

*OCI-R Ordering*

The regression model predicting OCI-R Ordering scores was significant (*R^2^*=.05, *p*<.001). Inspection of the individual regression coefficients revealed that the ECS was a significant individual predictor (*b=*.07 [*SE*=.01]*, p<*.001). Time per day consuming articles about COVID-19 (*b=*.21 [*SE*=.14]*, p*=.13), time per day using social media (*b=*.09 [*SE*=.14]*, p*=.52) and participant gender (*b=-*.19[*SE*=.45]*, p*=.67) were not significant predictors.

Moderation analysis found that the interaction term for ECS X daily hours of social media use was not significant (*b=*.01[*SE*=.01]*, p*=.48). Similarly, the interaction term for ECS X daily consumption of media pertaining to COVID-19 was also not significant (*b=*.02[*SE*=.01]*, p*=.17).

*OCI-R Neutralizing*

The regression model predicting OCI-R Neutralizing scores was significant (*R^2^*=.05, *p*<.001). Inspection of the individual regression coefficients revealed that the ECS was a significant individual predictor (*b=*.05 [*SE*=.01]*, p<*.001), as was time per day consuming articles about COVID-19 (*b=*.32 [*SE*=.11]*, p*<.01). Time per day using social media (*b=*.01 [*SE*=.11]*, p*=.91) and participant gender (*b=-*.30 [*SE*=.35]*, p*=.40) were not significant predictors.

Moderation analysis found that the interaction term for ECS X daily hours of social media use was not significant (*b=*.01[*SE*=.01]*, p*=.53). However, the interaction term for ECS X daily consumption of media pertaining to COVID-19 was significant (*b=*.04[*SE*=.01]*, p*<.001). For individuals low in emotion contagion (-1 SD on the ECS), there was not a significant effect between COVID-19 related media consumption and OCI-R Neutralizing scores (*b=*-.09[*SE*=.14]*, p*=.54). At the mean level of the ECS the effect was significant (*b=*.28[*SE*=.11]*, p*<.01), while the strength of this relationship was strongest for individuals at high in emotion contagion (+ 1 SD), (*b=*.69[*SE*=.14]*, p*<.001).

*OCI-R Washing*

The regression model predicting OCI-R washing scores was significant (*R^2^*=.08, *p*<.001). Inspection of the individual regression coefficients revealed that the ECS was a significant individual predictor (*b=*.07 [*SE*=.01]*, p<*.001), as was time per day consuming articles about COVID-19 (*b=*.30 [*SE*=.12]*, p*=.01). Time per day using social media (*b=*.14 [*SE*=.12]*, p*=.23) and participant gender (*b=*.01 [*SE*=.38]*, p*=.99) were not significant predictors.

Moderation analysis found that the interaction term for ECS X daily hours of social media use was not significant (*b=*.01[*SE*=.01]*, p*=.84). However, the interaction term for ECS X daily consumption of media pertaining to COVID-19 was significant (*b=*.03[*SE*=.01]*, p*=.004). For individuals low in emotion contagion (-1 SD on the ECS), there was not a significant effect between COVID-19 related media consumption and OCI-R Washing scores (*b=*.01[*SE*=.16]*, p*=.99). At the mean level of the ECS the effect was significant (*b=*.28[*SE*=.12]*, p*=.016), while the strength of this relationship was strongest for individuals at high in emotion contagion (+ 1 SD), (*b=*.59[*SE*=.15]*, p*<.001).

*OCI-R Obsessing*

The regression model predicting OCI-R washing scores was significant (*R^2^*=.07, *p*<.001). Inspection of the individual regression coefficients revealed that the ECS was a significant individual predictor (*b=*.07 [*SE*=.01]*, p<*.001), as was time per day consuming articles about COVID-19 (*b=*.35 [*SE*=.14]*, p*=.01) and time per day using social media (*b=*.30 [*SE*=.14]*, p*=.03). Participant gender (*b=*-.16[*SE*=.44]*, p*=.72) was not a significant predictor.

Moderation analysis found that the interaction term for ECS X daily hours of social media use was significant (*b=*.02[*SE*=.01]*, p*=.032). Specifically, for individuals low in emotion contagion (-1 SD on the ECS), there was not a significant effect between daily social media use and OCI-R Obsessing scores (*b=*.13[*SE*=.17]*, p*=.46). At the mean level of the ECS the effect was significant (*b=*.35 [*SE*=.13]*, p*=.008), while the strength of this relationship was strongest for individuals at high in emotion contagion (+ 1 SD), (*b=*.60[*SE*=.18]*, p*<.001). The interaction term for ECS X daily consumption of media pertaining to COVID-19 was not significant (*b=*.02[*SE*=.01]*, p*=.14).
